# Prevalence and Outcomes of Pancreatic Enzymes Elevation in Patients With COVID-19: A Meta-Analysis and Systematic Review

**DOI:** 10.3389/fpubh.2022.865855

**Published:** 2022-05-12

**Authors:** You Zhou, Yu-Tong Ge, Xiao-Xi Yang, Qian Cai, Yan-Bing Ding, Liang-Hao Hu, Guo-Tao Lu

**Affiliations:** ^1^Pancreatic Center, Department of Gastroenterology, Affiliated Hospital of Yangzhou University, Yangzhou University, Yangzhou, China; ^2^Yangzhou Key Laboratory of Pancreatic Disease, Institute of Digestive Diseases, Affiliated Hospital of Yangzhou University, Yangzhou University, Yangzhou, China; ^3^Department of Nursing, Affiliated Hospital of Yangzhou University, Yangzhou University, Yangzhou, China; ^4^Department of Oncology, Nanjing First Hospital, Nanjing Medical University, Nanjing, China; ^5^Department of Gastroenterology, Changhai Hospital, The Second Military Medical University, Shanghai, China

**Keywords:** COVID-19, pancreatic enzymes, elevation, outcome, meta-analysis, review

## Abstract

**Background:**

Although coronavirus disease 2019 (COVID-19) is considered to be a disease that mainly involves the respiratory system, an increasing number of studies have reported that COVID-19 patients had pancreatic enzymes (PE) elevation and even pancreatic injury. The study aims to determine the prevalence of PE elevation, and the relationship between elevated PE and prognosis in COVID-19 patients.

**Methods:**

A comprehensive literature search was conducted according to the PRISMA guideline in PubMed, Embase, Scopus, Web of Science, and Google Scholar for studies reporting PE elevation in patients with COVID-19 from 1st January 2020 to 24th November 2021.

**Results:**

A total of 13 studies (24,353 participants) were included in our review. The pooled prevalence of PE elevation in COVID-19 patients was 24% (18%–31%), the pooled odds ratio (OR) of mortality was 2.5 (1.7–3.6), the pooled OR of ICU admission was 4.4 (2.8–6.8), and the pooled OR of kidney injury, respiratory failure and liver injury were 3.5 (1.6–7.4), 2.0 (0.5–8.7), and 2.3 (1.4–3.9) respectively. In addition, the subgroup analysis revealed that although PE elevated to > 3 × upper normal limit (ULN) was significantly related to the mortality (OR = 4.4, 2.1–9.4), it seemed that mild elevation of PE to 1–3 ULN also had a considerable risk of mortality (OR = 2.3, 1.5–3.5).

**Conclusions:**

PE elevation was a common phenomenon in patients with COVID-19, and was associated with poor clinical outcomes. However, due to the limited numbers of included studies, the result of our study still needed to be validated.

**Systematic Review Registration:**

https://www.crd.york.ac.uk/PROSPERO/display_record.php?RecordID=295630, identifier: CRD42021295630.

## Introduction

Coronavirus disease 2019 (COVID-19) is a novel severe respiratory infectious disease caused by severe respiratory syndrome coronavirus-2 (SARS-CoV-2). Since the first case was officially reported in Wuhan, China in December 2019, COVID-19 has experienced a widespread outbreak and epidemic worldwide, which has caused tremendous impact and pressure on the medical and health systems around the world (1). On March 11, 2020, the World Health Organization announced it as a global pandemic disease. As of November 28, 2021, over 260 million confirmed cases have been reported globally, of which nearly 5.2 million died (2). COVID-19 has now developed into a global health crisis.

Although SARS-CoV-2 was believed to mainly invade the respiratory system of patients, with clinical manifestations as fever, cough, shortness of breath, and extensive lung consolidation, it cannot be ignored that some patients simultaneously had digestive symptoms as nausea, vomiting, and diarrhea (3–6). Consistent with SARS-CoV, SARS-CoV-2 invades cells through combining its spike protein with the angiotensin-converting enzyme II (ACE II) receptors (7, 8). Existing studies suggested that, in addition to type II alveolar epithelial cells, ACE II receptors are also highly expressed in esophagus, small intestine, colon and pancreas (9–11), and show a high affinity for SARS-CoV-2. Therefore, the pancreas may also be a potential target of SARS-CoV-2, which can lead to undetectable pancreatic injury (11).

Wang et al. (12) first reported pancreatic enzymes (PE) elevation in COVID-19 patients in a study involving infected people in the early stage of the epidemic. Subsequently, an increasing number of studies reported the similar findings. Since the critically ill COVID-19 patients often experience severe systemic inflammatory, shock, microcirculatory disturbance and renal failure, some scholars believed that PE elevation might be associated with pancreatic ischemic injury (13–15), and the elevated PE can serve as a surrogate marker for poor prognosis of COVID-19 patients. However, in different studies, due to the different sample sizes and definition of PE elevation, the prevalence of PE elevation varied greatly, and the clinical significance of it was still controversial (14, 16). In a previous meta-analysis by Goyal et al. (17), hyperlipasemia was found to be associated with the severity of COVID-19. However, in their study, severe COVID-19 was defined as death, intensive care unit (ICU) admission and need for mechanical ventilation, which was not rigorous because the elevated PE may have different impacts on different clinical outcomes. In addition, the included studies in their meta-analysis included letter to editor and correspondence, lacking enough case-control and cohort studies covering large samples and multi-centers. We believed that the result of their study was open to question.

Therefore, we performed this meta-analysis and systematic review in order to 1) determine the prevalence of PE elevation in COVID-19 patients, and 2) summarize the impact of PE elevation on the clinical outcomes in patients with COVID-19.

## Methods

### Protocol Registration

This meta-analysis and systematic review was conducted in accordance with the Preferred Reporting Items for Systematic Reviews and Meta-Analyses guidelines (18), and this study was part of the registered protocol on the International Prospective Register of Systematic Reviews (CRD42021295630).

### Search Strategy

With the assistance of a professional librarian, we determined the search terms and conducted a literature search in five online databases (PubMed, Embase, Scopus, Web of Science, and Google Scholar) from 1st January 2020 to 24th November 2021 for studies reporting PE elevation in COVID-19 patients. The literature search was limited to English publications. Search terms in PubMed included: [(“COVID-19”[MeSH] OR “COVID-19” OR “COVID 19” OR “COVID-19 Virus Disease^*^” OR “COVID-19 Virus Infection^*^”) OR (“SARS-CoV-2"[MeSH]” OR “SARS-CoV-2” OR “SARS-CoV-2 Virus^*^” OR “2019-nCoV” OR “Severe Acute Respiratory Syndrome Coronavirus 2”) OR (“Coronavirus”[Mesh] OR “Coronavirus” OR “Coronaviruses”)] AND [(“Amylases”[Mesh] OR “Amylases” OR “Amylase” OR “hyperamylasemia”) OR (“Lipase”[MeSH] OR “Lipase” OR “Hyperlipasemia”) OR (“pancreatic enzymes”)] AND (“elevat^*^”). Two reviewers (YZ and YTG) also screened the references of the key articles to include additional studies left out in the initial search.

### Eligibility Criteria

Based on the PICOS (Population, Intervention/Exposure, Comparison, Outcome, and Study design) strategy, the inclusion criteria were as follows:

Population: participants included in studies were clearly diagnosed with COVID-19.

- Exposure: PE elevation.
- Comparison: normal level of PE.
- Outcome: COVID-19 clinical outcomes (mortality or hospitalization or complications).
- Study design: Observational studies.

The exclusion criteria were as follows:

- Non-adult studies.
- Studies with unavailable full-text.
- Studies with unclear criteria for PE elevation.
- Studies not providing specific prevalence or outcomes of PE elevation.
- Protocols, review articles, abstracts, letters to editor, correspondence, case reports, and pre-prints.

### Study Selection

All identified articles were first imported into the Endnote X9 software to remove duplicates manually, then the titles and abstracts of studies were screened by two reviewers (XXY and QC) blindly in accordance with the inclusion and exclusion criteria to exclude irrelevant articles. The articles meeting the eligibility criteria were next screened on full text by the same two reviewers. Any disagreements were resolved by consulting another reviewer (YZ).

### Data Extraction

Data were extracted by two reviewers (YZ and XXY) using a designed Excel sheet. Any disagreements were solved by another reviewer (LHH). The following information was recorded: 1) author, 2) year of publication, 3) country, 4) study type, 5) samples size, 6) type of elevated PE, 7) definition for PE elevation, 8) proportion of patients with PE elevation among all patients, 9) proportion of patients with acute pancreatitis (AP) among patients with PE elevation, 10) clinical outcomes of COVID-19 patients with PE elevation.

### Quality Assessment

The Quality in Prognostic Studies tool was used to assess the quality of the included studies, which includes six items: study participation, study attrition, prognostic factor measurement, outcome measurement, confounding measurement and account, and analysis (19). Each article was assessed by two reviewers (YZ and QC) independently using a consistent standard. Any disagreements were resolved by consulting another reviewer (GTL).

### Statistical Analysis

The statistical analysis was performed using the Stata SE Version 16 software. We conservatively used a random-effects model to analyze the impact of PE elevation on mortality, ICU admission, and complications. A forest plot was used to visualized the data. The heterogeneity of included studies was estimated using the Cochran's Q-test and I^2^ statistics, and the value of I^2^ between 0 and 25%, 25–75%, and >75% was considered mild, moderate, and high heterogeneity, respectively (20). Prespecified subgroup analyses based on the definition of PE elevation and data source were performed to explore the heterogeneity of clinical outcomes between studies. Sensitivity analyses were preformed to explore the impact of each study by removing studies one by one. Egger's test and visual inspection of funnel plot were used to examine the publication bias. A *P*-value < 0.05 was considered statistically significant.

## Results

### Search Results

**Figure 1:**
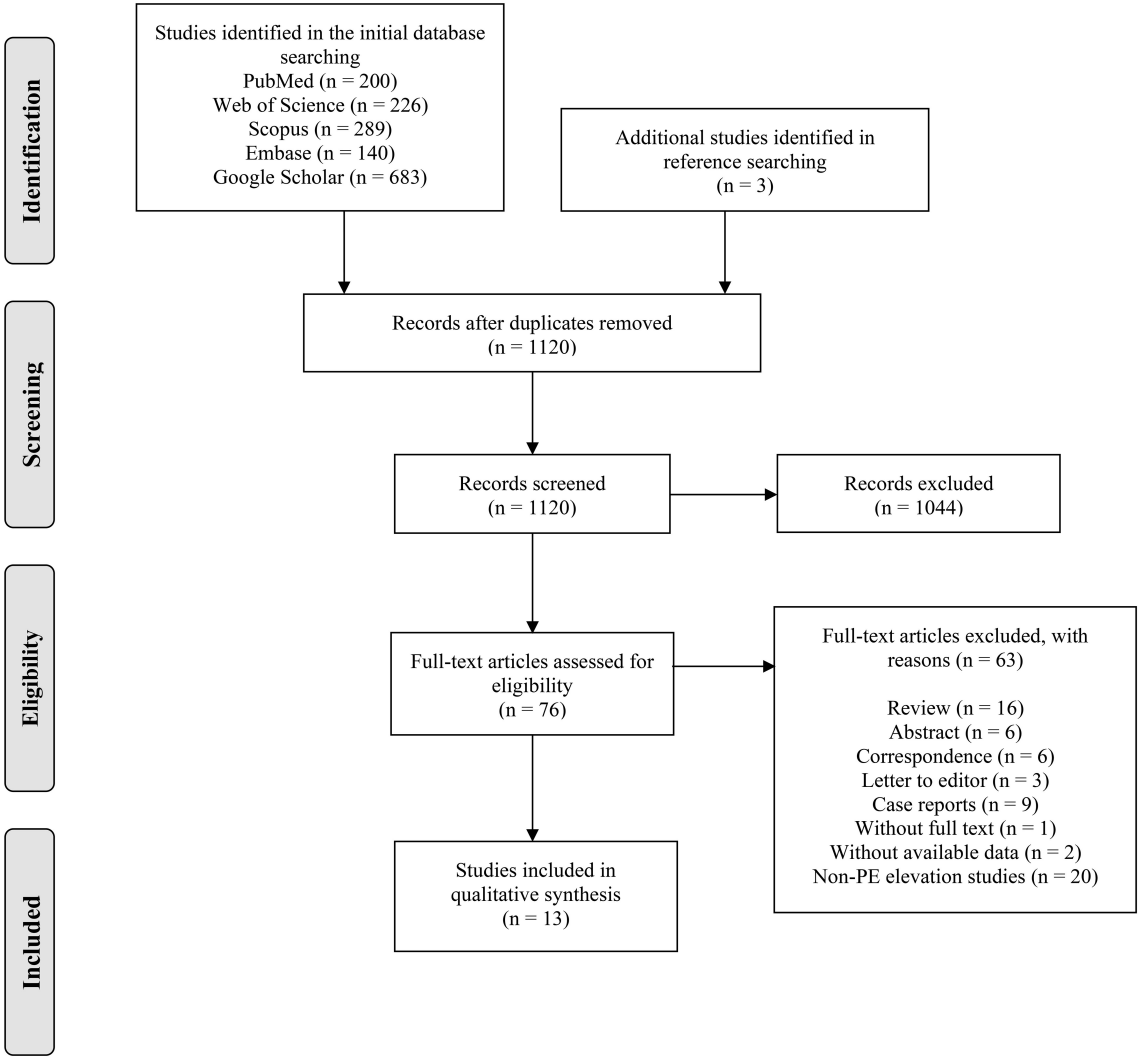
Preferred reporting items for systematic reviews and meta-analyses flow diagram.

The PRISAM flow diagram showed the process of article selection (Figure 1). A total of 1,538 records were extracted from the initial search, and three additional studies were identified through the reference searching. After removing the duplicates (*n* = 421), we screened 1,120 studies with titles and abstracts, of which 76 studies meeting the eligibility criteria were reviewed with full text. Thirteen studies (12–14, 16, 21–29) were finally included for qualitative and quantitative analysis in this review.

### Study Characteristics

Table 1 summarized the characteristic of the included studies. Thirteen studies were from the USA (*n* = 5), China (*n* = 3), Italy (*n* = 2), Turkey (*n* = 2), and Germany (*n* = 1), of which, 12 were retrospective, one (24) was prospective, and five studies (14, 16, 23, 25, 29) were multicenter. The sample size ranged from 38–17225, and the proportion of male participants varied from 44.6–78.9%. Each study had a clear definition of PE elevation, however, it lacked a unified standard and there was an obvious heterogeneity in the definition of upper normal limit (ULN). Ten studies (12–14, 16, 21–23, 26, 27, 29) defined PE elevation as > ULN, and three studies (24, 25, 28) defined PE elevation as > 3 × ULN. The results of quality assessment were shown in Supplementary Table 1.

**Table 1:** Characteristic of studies reporting pancreatic enzymes elevation in COVID-19 patients.

**Study**	**Year**	**Country**	**Study type**	**Male, *n* (%)**	**Age (mean ±SD)**	**Sample size, n**	**PE**	**Definition of PE elevation**
Ahmed et al. (14)	2021	USA	Retrospective	606 (61.1)	64 ± 17	992	Lipase	≥ ULN (Center 1: 78 IU/L, Center 2: 60 IU/L)
Bacaksiz et al. (13)	2021	Turkey	Retrospective	700 (51.8)	NP	1378	Amylase and lipase	≥ ULN (Amylase: 105 U/L, lipase: 65 U/L
Benias et al. (29)	2021	USA	Retrospective	680 (46.2%)	NP	1471	Lipase	≥ ULN
Caruso et al. (21)	2021	Italy	Retrospective	692 (63.4)	64 (IQR: 52–77)	1092	Lipase	≥ ULN (45 U/L)
Ding et al. (22)	2021	China	Retrospective	37 (67.3)	63 (Range: 29-79)	55	Amylase and lipase	≥ ULN (Amylase: 135 U/L, lipase: 78 U/L
Li et al. (23)	2021	China	Retrospective	737 (48.6)	61 (IQR: 49–69)	1515	Amylase	≥ ULN (115 U/L)
Rasch et al. (24)	2021	Germany	Prospective	30 (78.9)	68.5 (Range: 26–85)	38	Lipase	≥ 3 ULN (60 U/L)
Singh et al. (25)	2021	USA	Retrospective	8349 (52.7)	NP	17225	Lipase	≥ 3 × ULN or 180 U/L
Troncone et al. (26)	2021	Italy	Retrospective	148 (58.3)	67 (IQR: 53–81)	254	Amylase and lipase	≥ ULN (Amylase: 125 U/L for patients <70 years old, 160 U/L for patients >70 years old; lipase: 78 U/L)
Akkus et al. (27)	2020	Turkey	Retrospective	73 (57.5)	NP	127	Lipase	≥ ULN (60 U/L)
Baltar et al. (16)	2020	USA	Retrospective	33 (46.5)	69.4 ± 15.8	71	Lipase	≥ ULN (60 U/L)
Barlass et al. (28)	2020	USA	Retrospective	37 (44.6)	NP	83	Lipase	≥ 3 × ULN (52 U/L)
Wang et al. (12)	2020	China	Retrospective	24 (46.2)	NP	52	Amylase and lipase	≥ ULN (Amylase: 90 U/L, lipase: 70 U/L)

*NP, not reported; PE, pancreatic enzymes, ULN: upper normal limit*.

### Prevalence of PE and AP

**Figure 2:**
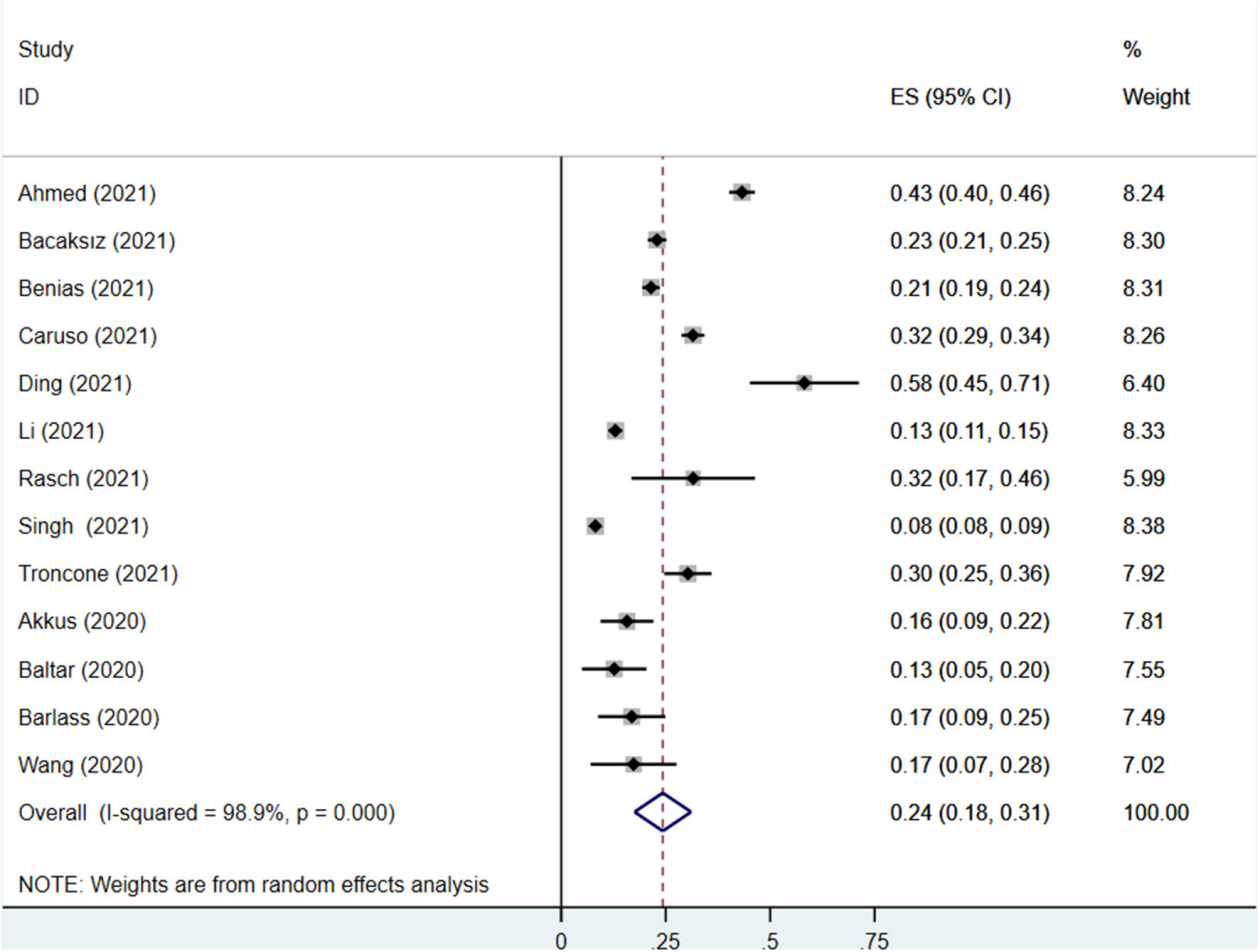
Effect size analysis for the prevalence of PE elevation in COVID-19 patients.

Thirteen studies reported the prevalence of PE elevation in COVID-19 patients, covering 2,4353 participants, of which 3,180 participants had elevated PE. The prevalence of PE elevation ranged from 8.2 to 58.2%. The pooled prevalence of PE elevation in COVID-19 patients was 24% (95% CI: 18%−31%), with a high degree of heterogeneity (I^2^ = 98.9%) (Figure 2).

Six studies (13, 14, 21, 22, 25, 26) with samples more than ten patients reported AP diagnosis according to the revised Atlanta classification of acute pancreatitis 2012 (30), covering 1,705 patients with elevated PE > 3 × ULN, of which 182 developed AP. The prevalence of AP ranged from 1.3 to 18.8%. The pooled prevalence of AP in patients with elevated PE > 3 × ULN was 9% (95% CI: 2%-15%), with a high degree of heterogeneity (I^2^= 93.2%) (Supplementary Figure 1).

### Analysis of PE Elevation and Mortality

**Figure 3:**
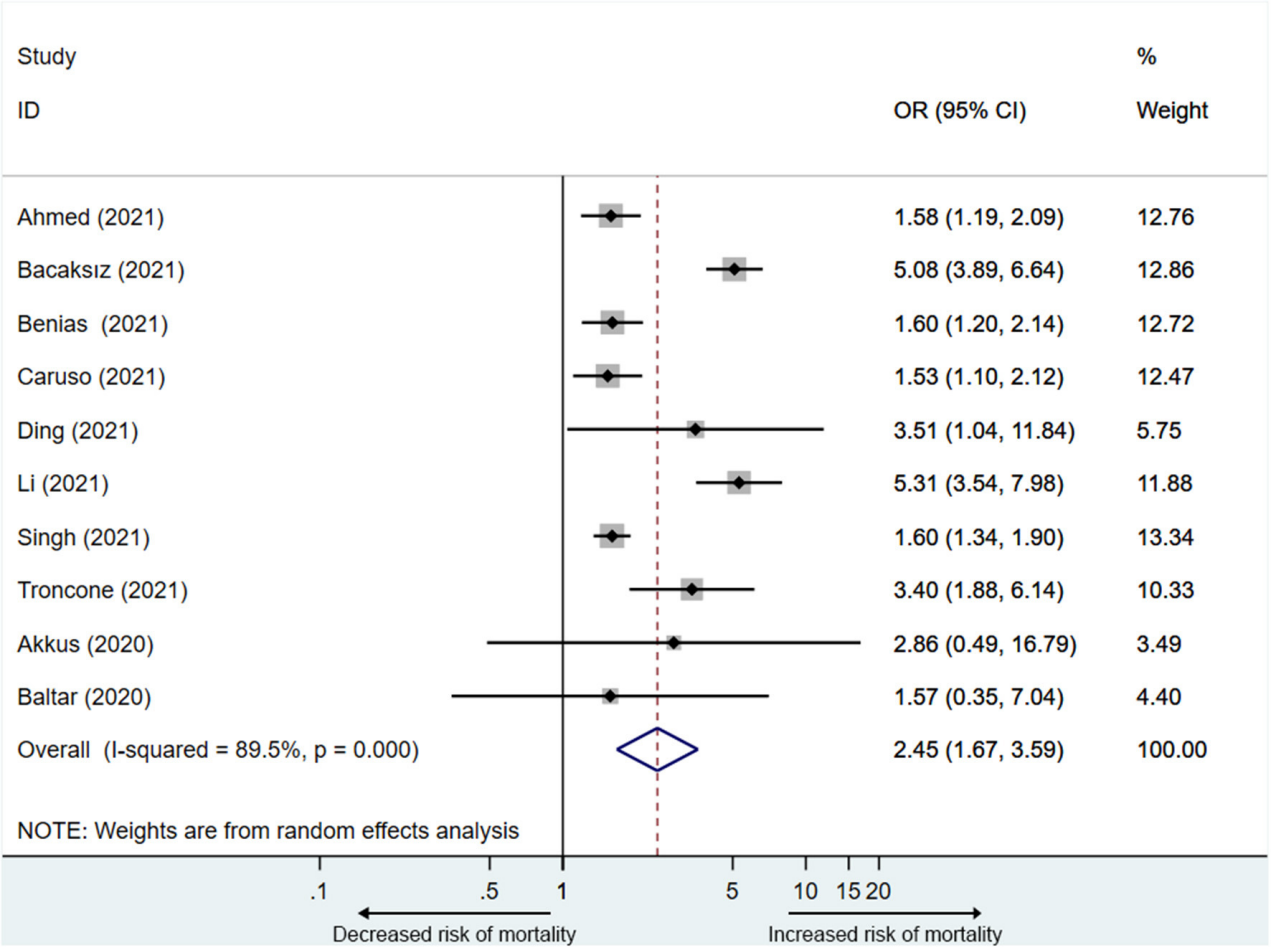
Effect size analysis for mortality in COVID-19 patients with PE elevation.

Ten studies (13, 14, 16, 21–23, 25–27, 29) reported the mortality associated with elevated PE. A total of 2,4207 participants including 3,142 participants in the elevated PE group, of which 760 participants died, and 2,1065 participants in the normal PE group, of which 2,033 participants died were included in the analysis. The mortality ranged from 10.0 to 79.3%. PE elevation was significantly related to the mortality of COVID-19 patients (OR = 2.5, 95% CI: 1.7–3.6), with substantial heterogeneity (I^2^ = 89.5%) (Figure 3).

Since the heterogeneity was significant, we performed a sensitivity analysis to explore the impact of each study. The result showed that two studies (13, 23) affected the pooled OR (odds ratio) of mortality (Supplementary Figure 2). After removing any one of the two studies, the I^2^ did not decrease significantly (78.6–87.7%). After removing both studies simultaneously, the heterogeneity became acceptable (I^2^ = 13.3%), and the pooled OR was 1.7 (95% CI: 1.5–1.9) (Supplementary Figure 3).

Subsequently, we performed a subgroup analysis based on the definition of PE elevation and data sources. Ten studies were divided into the 1–3 ULN group (13, 14, 21, 23, 26, 29) and the >3 × ULN group (13, 14, 21, 23, 25, 26, 29) (six studies (13, 14, 21, 23, 26, 29) reported the two conditions). The 1–3 ULN group involved 1,330 participants, of which 424 died, and the >3 × ULN group involved 1,754 participants, of which 308 died. The result of subgroup analysis showed that PE elevated to both 1–3 ULN (OR= 2.3, 95% CI: 1.5–3.5) and >3 × ULN (OR = 4.4, 95% CI: 2.1–9.4) were significantly related to mortality, and the pooled OR of the 1–3 ULN group was similar to that before grouping (OR = 2.5, 95% CI: 1.7–3.6) (Supplementary Figure 4). Considering two studies with obvious heterogeneity, we also performed subgroup analysis after removing these two studies (Table 2) (Supplementary Figure 4). Consistent with the previous result, after removing the heterogeneous studies, the pooled OR of the 1–3ULN group (OR = 1.7, 95% CI: 1.3–2.1) was basically the same as that of all eight studies (OR = 1.7, 95% CI: 1.5–1.9).

**Table 2:** Subgroup analysis on the association between of pancreatic enzymes elevation and mortality in COVID-19 patients.

**Subgroups**	**Number of studies**	**Sample size (n)**	**OR**	**95% CI**	**I^**2**^ (%)**	***P*-value**
**Definition of PE elevation**						
1–3 ULN	6 (13, 14, 21, 23, 26, 29)	1330	2.3	1.5–3.5	87.5	<0.001
>3 ULN	7 (13, 14, 21, 23, 25, 26, 29)	1754	4.4	2.1–9.4	92.9	<0.001
Reference	10	3142	2.5	1.7–3.6	89.5	<0.001
1–3 ULN^*^	4 (14, 21, 26, 29)	892	1.7	1.3–2.1	41.1	0.165
>3 ULN^*^	5 (14, 21, 25, 26, 29)	1680	1.9	1.3–2.6	61.1	0.036
Reference^*^	8	2630	1.7	1.5–1.9	13.3	0.326
**Data source**						
Single-center^*^	4 (21, 22, 26, 27)	470	2.4	1.4–4.2	54.4	0.087
Multi-center^*^	4 (14, 16, 25, 29)	3554	1.6	1.4–1.8	0.0	1.000
Reference^*^	8	2630	1.7	1.5–1.9	13.3	0.326

* *After removing the two heterogeneous studies (13, 23)*.

*CI, confidence interval; OR, odds ratio; PE, pancreatic enzyme; ULN, upper normal limit*.

Based on the different sources of data and removing the heterogeneous studies, we categorized eight studies into the single-center group (21, 22, 26, 27) and the multi-center group (14, 16, 25, 29). Compared with the pooled OR of all eigth studies (OR = 1.7, 95% CI: 1.5–1.9) and four multi-center studies (OR= 1.6, 95% CI: 1.4–1.8), it was worth noting that the pooled OR of single-center group seemed to be higher (OR = 2.4, 95% CI: 1.4–4.2) (Table 2) (Supplementary Figure 4).

### Analysis of PE Elevation and Hospitalization

**Figure 4:**
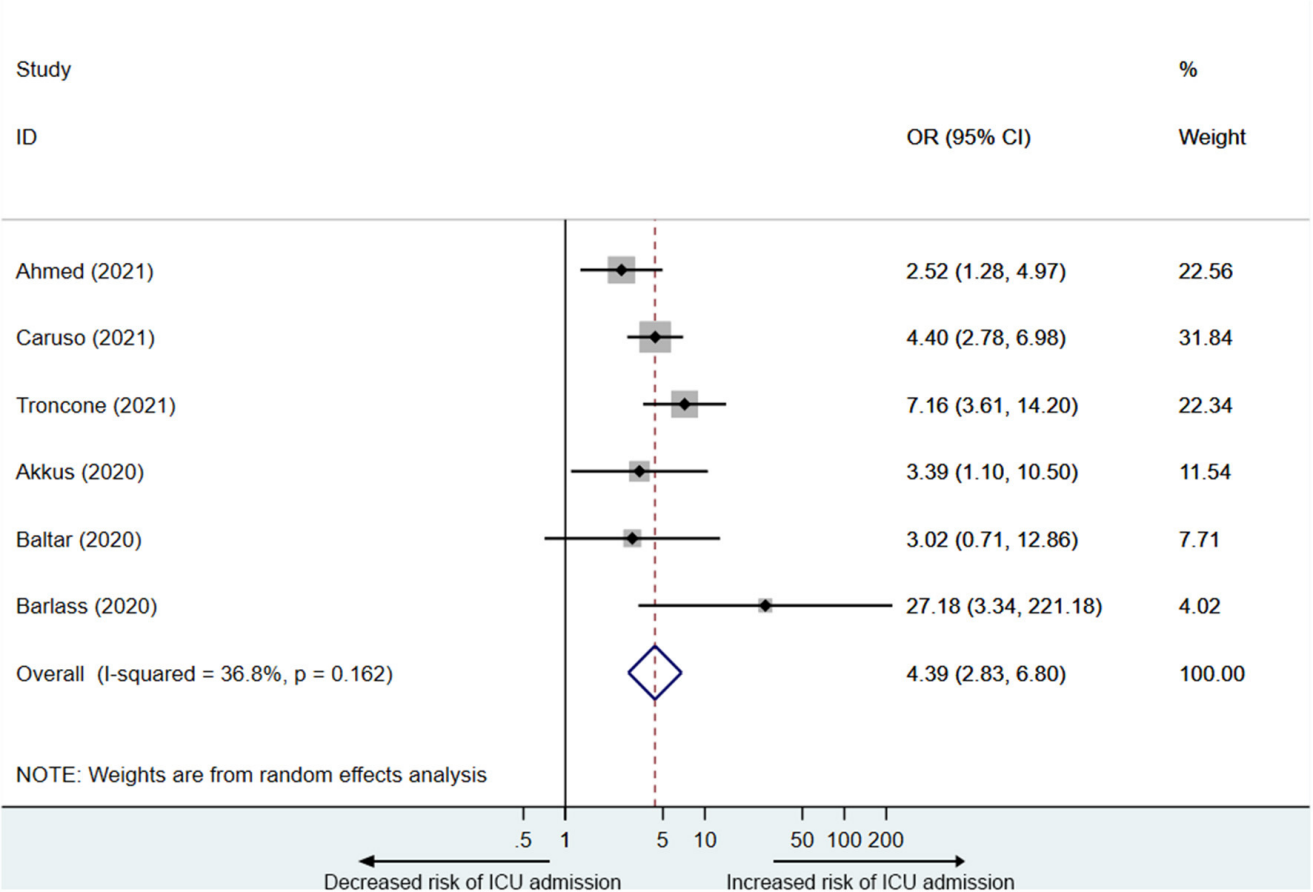
Effect size analysis for ICU admission in COVID-19 patients with PE elevation.

Six studies (14, 16, 21, 26–28) reported PE elevation was associated with ICU admission in COVID-19 patients. A total of 1,783 participants including 520 participants in the elevated PE group, of which 147 were admitted to the ICU, and 1,263 participants in the normal PE group, of which 138 were admitted to the ICU were included in the analysis. As is shown in Figure 4, PE elevation was significantly associated with ICU admission in COVID-19 patients (OR = 4.4, 95% CI: 2.8–6.8), with acceptable heterogeneity (I^2^ = 36.8%).

Three studies reported that PE elevation was related to the length of stay (LOS). Ahmed et al. (14) reported that the median LOS for patients with elevated PE was 15 days (IQR: 8.3–30 days), and that for patients with normal PE was 11 days (IQR: 5.5–20.5 days). Akkus et al. (27) found that the median LOS for patients with elevated PE was 11.5 days (range: 3–41 days), and that for patients with normal PE was 8 days (range: 0–38 days). Benias et al. (29) reported that the LOS for patients with normal PE, 1–3 ULN PE, and >3 × ULN PE was 11.19, 15.08, and 24.20 days respectively. Compared with normal PE, the median LOS for COVID-19 patients with elevated PE increased by about 40%.

In addition, Ahmed et al. (14) found that patients with elevated PE had longer ICU LOS. Compared with those without PE elevation (12days, IQR: 3.3–20 days), the median ICU LOS for patients with elevated PE (19 days, IQR: 7.5–33.5 days) increased by about 60%. Ding et al. (22) (20.7% vs. 47.8%) and Li et al. (23) (76.5% vs. 94.5%) found that patients with elevated PE had a lower discharge rate respectively. Singh et al. (25) reported that patients with or without PE elevation did not show significant difference in rehospitalization (42.0% vs. 42.8%).

### Analysis of PE Elevation and Complications

Six studies (13, 22–26) reported complications in PE elevation patients. Among them, kidney injury (KI) was the most common complication. The meta-analysis suggested that elevated PE was significantly associated with the increased risk of KI (OR= 3.5, 95% CI: 1.6–7.4), with significant heterogeneity (I^2^ = 95.0%) (Supplementary Figure 5). There were two studies each reporting respiratory failure (23, 25) and liver failure (24, 26). The pooled OR of respiratory failure in COVID-19 patients with elevated PE was 2.0 (95% CI: 0.5–8.7) (Supplementary Figure 5), and the pooled OR of liver failure was 2.3 (95% CI: 1.4–3.9) (Supplementary Figure 5). In addition, the reported complications included acute heart failure, cardiac injury, sepsis, and disseminated intravascular coagulation as well (23).

### Publication Bias

Egger's test revealed that, there was no significant publication bias for studies reporting mortality (*P* = 0.463), ICU admission (*P* = 0.647), and KI (*P* = 0.523) associated with PE elevation, except for PE prevalence (*P* = 0.006). Supplementary Figure 6 for visual funnel plots.

## Discussion

To the best of our knowledge, this is the latest and most comprehensive systematic review and meta-analysis on the prevalence and clinical outcomes of PE elevation in COVID-19 patients. Our study demonstrated that, overall, PE elevation was common in COVID-19 patients. The pooled prevalence of PE elevation was 24%, which was significantly higher than that of the previous mate-analysis by Goyal et al., and the risk of developing severe COVID-19 in patients with hyperlipasemia in their study was higher than that of adverse outcomes in our study (17). This is understandable because their meta-analysis included fewer and earlier studies. In addition, we also found that about 9% of patients with elevated PE > 3 × ULN eventually developed AP, which was also higher than that of a previous meta-analysis on the prevalence and clinical outcomes of AP in COVID-19 patients reported by Yang et al. (31). In their study, the pooled prevalence of AP complicated by COVID-19 was about 3.1%, of which about 18.5% eventually died. COVID-19 patients with pancreatic injury often had poor clinical outcomes. According to the revised Atlanta classification of acute pancreatitis 2012, the diagnosis of AP included abdominal pain, the elevated PE > 3 × ULN, and characteristic findings of AP on imaging. However, it cannot be denied that patients did not meet the diagnostic have no potential pancreatic injury and potential risk of poor prognosis. In this systematic review and meta-analysis, we extensively searched and included existing studies on PE elevation in patients with COVID-19 and included more participants to reveal the association between elevated PE and the clinical outcomes of COVID-19 patients.

At present, the cause of PE elevation was still unclear. In the autopsy of patients with severe acute respiratory syndrome, SARS-CoV was found to be present in pancreatic tissue (32). Due to the similarity of the two viruses and the ACE II receptors highly expressed in the pancreas, pancreatic injury caused by the direct invasion of SARS-CoV-2 was one of the potential causes of PE elevation. In the case report by Schepis et al., SARS-CoV-2 RNA was detected for the first time in a pancreatic pseudocyst fluid sample from a COVID-19 patient (33). In addition, a Chinese pathology study found that COVID-19 patients had a small amount of pancreatic islet cell degeneration (34). Although the above studies seemed to verify the possibility of direct damage by SARS-CoV-2, in critically ill patients, PE elevation often occurred. The most widely accepted explanation for PE elevation with non-viral causes was pancreatic ischemia (15, 35, 36). When the patient had severe infection, hypoperfusion and shock, the pancreas was insufficiently perfused, which will lead to pancreatic injury. In addition, non-pancreatic causes such as intestinal inflammation (37), diabetes (38), acidosis (39), and renal failure (38, 40, 41) can also lead to PE elevation. Although a variety of causes, including pancreatic injury, can lead to PE elevation, it was undeniable that when the above symptoms appeared in COVID-19 patients, it often indicated the occurrence of poor clinical outcomes.

Our meta-analysis and systematic review found that PE elevation in COVID-19 patients was significantly associated with the increase of mortality, ICU admission, LOS, and clinical complications as KI, respiratory failure and liver failure. In the analysis of mortality, the pooled OR without two heterogeneous studies (13, 23) was 1.7, which was significantly lower than the pooled OR of 2.5 for all studies. In these two studies, we found that 50.1% of the patients were diagnosed with severe COVID-19 and 43% with severe pneumonia, respectively. In a meta-analysis involving 30 studies, the proportion of severe COVID-19 was about 26% (42). In addition, studies have shown that severe COVID-19 and more comorbidities were the risk factors for higher clinical mortality (43, 44). Although the severity of COVID-19 was not clearly reported in other included studies, we believed that the heavier condition of patients contributed to the higher mortality, resulting in the overestimation of the risk of pooled mortality. Compared with the existing discovered risk factors for mortality of COVID-19 patients such as gender (45, 46), age (43, 45–47), diabetes (45, 48), history of COPD (45), and chronic cardiac disease (49), we found that PE elevation had a similar risk for mortality. Therefore, PE elevation may also serve as a risk indicator of mortality for patients with COVID-19.

In the subgroup analysis of mortality based on different definition of PE elevation, we found that patients with elevated PE of >3 × ULN had a higher risk of death. In addition, it was also interesting that regardless of including or excluding the heterogeneous studies, the pooled OR of mortality in the 1–3 ULN group did not change a lot (2.3 vs. 2.5, 1.7 vs. 1.7), which indicated that a slight increase in PE, even if it did not satisfy the diagnostic criterion of AP, will have a hazardous effect on the clinical outcomes of COVID-19 patients. In other words, it is possible that PE is a sensitive marker for predicting the mortality in COVID-19 patients.

In the subgroup analysis on mortality based on different data sources, the pooled OR of mortality in single-center studies was higher than that of multi-center studies (2.4 vs. 1.6). Among the included multi-center studies, one was a database study (25), one study was based on two tertiary hospitals and four community hospitals (16), and two studies was based on several major tertiary medical systems (14, 29). Since the time of data recorded and the methods of measurement and testing in public databases were difficult to ensure consistency (50), and the conditions of patients in community hospitals were different from those in tertiary medical institutions, we believed that the existing multi-center studies may underestimate the real risk of mortality in COVID-19 patients with elevated PE. Therefore, we hoped that prospective studies based on several tertiary medical institutions can be carried out to explore the real risk of hospital mortality related to PE elevation in COVID-19 patients. And on this basis, further explore the specific sources and risk factors of PE elevation.

In addition, it is worth noting that Ahmed et al. (14) tried to explore the relationship between PE elevation, D-dimer and mortality, ICU admission. Existing studies have proved that laboratory factors including D-dimer levels, demographic factors, patient history factors, physical examination factors, and clinical scores were significantly related to the severity and poor prognosis of COVID-19 patients (51). Since the COVID-19 patients often underwent various examinations during hospitalization, which generated rich medical records, it will be a meaningful attempt to predict the clinical outcomes of COVID-19 patients through using these multi-dimensional data. At present, machine learning algorithm has been widely used in the prediction tasks of complications, mortality, etc. in COVID-19 (52–54). We hoped that future studies can develop similar predictive models based on multi-omics clinical data including PE elevation to predict the clinical outcome of COVID-19 patients.

This systematic review and meta-analysis also had certain limitations. First, we only searched articles in English, which may lead to potential bias of publication. Second, due to the limited number of articles included, the results showed significant heterogeneity. Although we attributed it to the differences in the severity of COVID-19, there may also be other potential factors that we overlooked. Third, although we tried to perform a subgroup analysis to explore the impact of PE elevation on specific clinical outcomes, due to the few studies reporting detailed complications, the result of our analysis was unstable and needed to be validated by including more studies in the future.

## Conclusion

In conclusion, our research found that PE elevation was a risk factor for poor clinical outcomes in patients with COVID-19. Compared with patients with normal PE, patients with elevated PE had a higher risk of mortality, ICU admission, and complications. In addition, future studies are still needed for further analysis of more impacts of PE elevation in COVID-19 patients.

## Data Availability Statement

The raw data supporting the conclusions of this article will be made available by the authors, without undue reservation.

## Author Contributions

All authors contributed to the development of the manuscript. YZ, Y-TG, L-HH, and G-TL designed the study. YZ and Y-TG conducted literature searching with the help of Y-BD and L-HH. X-XY and QC screened and reviewed the articles. YZ and QC assessed the quality of included studies. YZ and X-XY extracted the data from included studies. YZ and Y-TG drafted the manuscript. Y-BD, L-HH, and G-TL provided guidance and approved the final draft. All authors contributed to the article and approved the submitted version.

## Funding

This study was supported by the National Natural Science Foundation of China [No. 82070664 (L-HH), No. 81801970 (G-TL), and No. 82070668 (G-TL)].

## Conflict of Interest

The authors declare that the research was conducted in the absence of any commercial or financial relationships that could be construed as a potential conflict of interest.

## Publisher's Note

All claims expressed in this article are solely those of the authors and do not necessarily represent those of their affiliated organizations, or those of the publisher, the editors and the reviewers. Any product that may be evaluated in this article, or claim that may be made by its manufacturer, is not guaranteed or endorsed by the publisher.

## References

[1] Zhu N Zhang D Wang W Li X Yang B Song J . A novel coronavirus from patients with pneumonia in China, 2019. N Engl J Med. (2020) 382:727–33. 10.1056/NEJMoa2001017 31978945 PMC7092803

[2] World Health O. Covid-19 Weekly Epidemiological Update, Edition 68, 30 November 2021. Geneva: World Health Organization (2021).

[3] Chen N Zhou M Dong X Qu J Gong F Han Y . Epidemiological and clinical characteristics of 99 cases of 2019 novel coronavirus pneumonia in Wuhan, China: a descriptive study. Lancet (London, England). (2020) 395:507–13. 10.1016/S0140-6736(20)30211-7 32007143 PMC7135076

[4] Huang C Wang Y Li X Ren L Zhao J Hu Y . Clinical features of patients infected with 2019 novel coronavirus in Wuhan, China. Lancet (London, England). (2020) 395:497–506. 10.1016/S0140-6736(20)30183-5 31986264 PMC7159299

[5] Mao R Qiu Y He J-S Tan J-Y Li X-H Liang J . Manifestations and prognosis of gastrointestinal and liver involvement in patients with Covid-19: a systematic review and meta-analysis. Lancet Gastroenterol Hepatol. (2020) 5:667–78. 10.1016/S2468-1253(20)30126-6 32405603 PMC7217643

[6] Jin X Lian J-S Hu J-H Gao J Zheng L Zhang Y-M . Epidemiological, clinical and virological characteristics of 74 cases of coronavirus-infected disease 2019 (Covid-19) with gastrointestinal symptoms. Gut. (2020) 69:1002–9. 10.1136/gutjnl-2020-320926 32213556 PMC7133387

[7] Zhang H Penninger JM Li Y Zhong N Slutsky AS. Angiotensin-converting enzyme 2 (Ace2) as a Sars-Cov-2 receptor: molecular mechanisms and potential therapeutic target. Intensive Care Med. (2020) 46:586–90. 10.1007/s00134-020-05985-9 32125455 PMC7079879

[8] Kuhn JH Li W Choe H Farzan M. Angiotensin-Converting enzyme 2: a functional receptor for Sars coronavirus. Cell Mol Life Sci. (2004) 61:2738–43. 10.1007/s00018-004-4242-5 15549175 PMC7079798

[9] Ma C Cong Y Zhang H. Covid-19 and the digestive system. Am J Gastroenterol. (2020) 115:1003–6. 10.14309/ajg.0000000000000691 32618648 PMC7273952

[10] Xu H Zhong L Deng J Peng J Dan H Zeng X . High expression of Ace2 receptor of 2019-Ncov on the epithelial cells of oral mucosa. Int J Oral Sci. (2020) 12:8. 10.1038/s41368-020-0074-x 32094336 PMC7039956

[11] Liu F Long X Zhang B Zhang W Chen X Zhang Z. Ace2 expression in pancreas may cause pancreatic damage after Sars-Cov-2 infection. Clin Gastroenterol Hepatol. (2020) 18:2128–30. 10.1016/j.cgh.2020.04.040 32334082 PMC7194639

[12] Wang F Wang H Fan J Zhang Y Wang H Zhao Q. Pancreatic injury patterns in patients with coronavirus disease 19 pneumonia. Gastroenterology. (2020) 159:367–70. 10.1053/j.gastro.2020.03.055 32247022 PMC7118654

[13] Bacaksiz F Ebik B Ekin N Kilic J. Pancreatic damage in Covid-19: why? How? Int J Clin Pract. (2021) 75:e14692. 10.1111/ijcp.14692 34331821 PMC8420122

[14] Ahmed A Fisher JC Pochapin MB Freedman SD Kothari DJ Shah PC . Hyperlipasemia in absence of acute pancreatitis is associated with elevated D-Dimer and adverse outcomes in Covid 19 disease. Pancreatology. (2021) 21:698–703. 10.1016/j.pan.2021.02.021 33741267 PMC7929790

[15] Chaari A Abdel Hakim K Bousselmi K Etman M El Bahr M El Saka A . Pancreatic injury in patients with septic shock: a literature review. World J Gastrointest Oncol. (2016) 8:526–31. 10.4251/wjgo.v8.i7.526 27559431 PMC4942740

[16] McNabb-Baltar J Jin DX Grover AS Redd WD Zhou JC Hathorn KE . Lipase elevation in patients with Covid-19. Am J Gastroenterol. (2020) 115:1286–8. 10.14309/ajg.0000000000000732 32496339 PMC7288768

[17] Goyal H Sachdeva S Perisetti A Mann R Inamdar S Tharian B. Hyperlipasemia and potential pancreatic injury patterns in Covid-19: a marker of severity or innocent bystander? Gastroenterology. (2021) 160:946–8. 10.1053/j.gastro.2020.10.037 33129845 PMC7598680

[18] Moher D Liberati A Tetzlaff J Altman DG. Preferred reporting items for systematic reviews and meta-analyses: the prisma statement. PLoS Med. (2009) 6:e1000097. 10.1371/journal.pmed.1000097 19621072 PMC2707599

[19] Hayden JA van der Windt DA Cartwright JL Côté P Bombardier C. Assessing bias in studies of prognostic factors. Ann Intern Med. (2013) 158:280–6. 10.7326/0003-4819-158-4-201302190-00009 23420236

[20] Nagarajan R Krishnamoorthy Y Basavarachar V Dakshinamoorthy R. Prevalence of post-traumatic stress disorder among survivors of severe Covid-19 infections: a systematic review and meta-analysis. J Affect Disord. (2022) 299:52–9. 10.1016/j.jad.2021.11.040 34800571 PMC8596764

[21] Caruso S Aloisio E Dolci A Panteghini M. Lipase Elevation in serum of Covid-19 patients: frequency, extent of increase and clinical value. Clin Chem Lab Med. (2021):60:135–42. 10.1515/cclm-2021-0824 34687597

[22] Ding P Song B Liu X Fang X Cai H Zhang D. et al. Elevated pancreatic enzymes in Icu patients with Covid-19 in Wuhan, China: a retrospective study. Front Med. (2021) 8:663646. 10.3389/fmed.2021.663646 34485322 PMC8415839

[23] Li G Liu T Jin G Li T Liang J Chen Q . Serum amylase elevation is associated with adverse clinical outcomes in patients with coronavirus disease 2019. Aging (Albany NY). (2021) 13:23442–58. 10.18632/aging.203653 34714255 PMC8580346

[24] Rasch S Herner A Schmid RM Huber W Lahmer T. High lipasemia is frequent in Covid-19 associated acute respiratory distress syndrome. Pancreatology. (2021) 21:306–11. 10.1016/j.pan.2020.11.023 33277183 PMC7700722

[25] Singh RR Chhabra P Kumta NA. Does hyperlipasemia predict worse clinical outcomes in Covid-19? a multicenter retrospective cohort study. J Clin Gastroenterol. (2021) 56:e227–231. 10.1097/MCG.0000000000001590 34294655 PMC8843055

[26] Troncone E Salvatori S Sena G De Cristofaro E Alfieri N Marafini I . Low frequency of acute pancreatitis in hospitalized Covid-19 patients. Pancreas. (2021) 50:393–8. 10.1097/MPA.0000000000001770 33835971

[27] Akkus C Yilmaz H Mizrak S Adibelli Z Akdas O. Duran C. Development of pancreatic injuries in the course of Covid-19. Acta Gastroenterol Belg. (2020) 83:585–92. 33321015

[28] Barlass U Wiliams B Dhana K Adnan D Khan SR Mahdavinia M . Marked elevation of lipase in Covid-19 disease: a cohort study. Clin Transl Gastroenterol. (2020) 11:e00215. 10.14309/ctg.0000000000000215 32764201 PMC7386395

[29] Benias PC Inamdar S Wee D Liu Y Buscaglia JM Satapathy SK . Analysis of outcomes in Covid-19 patients with varying degrees of hyperlipasemia. Pancreas. (2021) 50:1310–3. 34860817 10.1097/MPA.0000000000001922

[30] Banks PA Bollen TL Dervenis C Gooszen HG Johnson CD Sarr MG . Classification of acute pancreatitis−2012: revision of the Atlanta classification and definitions by international consensus. Gut. (2013) 62:102–11. 10.1136/gutjnl-2012-302779 23100216

[31] Yang F Huang Y Li T Fu Y Sun C Xu Y . Prevalence and outcomes of acute pancreatitis in Covid-19: a meta-analysis. Gut. (2021). 10.1136/gutjnl-2021-325941 34670809

[32] Ding Y He L Zhang Q Huang Z Che X Hou J . Organ distribution of Severe Acute Respiratory Syndrome (Sars) Associated Coronavirus (Sars-Cov) in Sars patients: implications for pathogenesis and virus transmission pathways. J Pathol. (2004) 203:622–30. 10.1002/path.1560 15141376 PMC7167761

[33] Schepis T Larghi A Papa A Miele L Panzuto F De Biase L . Sars-Cov2 Rna detection in a pancreatic pseudocyst sample. Pancreatology. (2020) 20:1011–2. 10.1016/j.pan.2020.05.016 32498972 PMC7254005

[34] Yao XH Li TY He ZC Ping YF Liu HW Yu SC . [A pathological report of three Covid-19 cases by minimal invasive autopsies]. Zhonghua Bing Li Xue Za Zhi. (2020) 49:411–7. 10.3760/cma.j.cn112151-20200312-00193 32172546

[35] Cohen J MacArthur KL Atsawarungruangkit A Perillo MC Martin CR Berzin TM . Defining the diagnostic value of hyperlipasemia for acute pancreatitis in the critically ill. Pancreatology. (2017) 17:176–81. 10.1016/j.pan.2017.02.005 28237616

[36] Hiltebrand LB Krejci V Banic A Erni D Wheatley AM Sigurdsson GH. Dynamic study of the distribution of microcirculatory blood flow in multiple splanchnic organs in septic Shock. Crit Care Med. (2000) 28:3233–41. 10.1097/00003246-200009000-00019 11008987

[37] Tositti G Fabris P Barnes E Furlan F Franzetti M Stecca C . Pancreatic hyperamylasemia during acute gastroenteritis: incidence and clinical relevance. BMC Infect Dis. (2001) 1:18. 10.1186/1471-2334-1-18 11667952 PMC58589

[38] Hameed AM Lam VWT Pleass HC. Significant elevations of serum lipase not caused by pancreatitis: a systematic review. HPB (Oxford). (2015) 17:99–112. 10.1111/hpb.12277 24888393 PMC4299384

[39] Yadav D Nair S Norkus EP Pitchumoni CS. Nonspecific hyperamylasemia and hyperlipasemia in diabetic ketoacidosis: incidence and correlation with biochemical abnormalities. Am J Gastroenterol. (2000) 95:3123–8. 10.1111/j.1572-0241.2000.03279.x 11095328

[40] Robitaille R Lafrance J-P Leblanc M. Altered laboratory findings associated with end-stage renal disease. Semin Dial. (2006) 19:373–80. 10.1111/j.1525-139X.2006.00192.x 16970737

[41] Muniraj T Dang S. Pitchumoni CS. Pancreatitis or not?–elevated lipase and amylase in Icu patients. J Crit Care. (2015) 30:1370–5. 10.1016/j.jcrc.2015.08.020 26411523

[42] Hu J Wang Y. The Clinical Characteristics and Risk Factors of Severe Covid-19. Gerontology. (2021) 67:255–66. 10.1159/000513400 33406518 PMC7900480

[43] Zhou F Yu T Du R Fan G Liu Y Liu Z . Clinical course and risk factors for mortality of adult inpatients with Covid-19 in Wuhan, China: a retrospective cohort study. Lancet (London, England). (2020) 395:1054–62. 10.1016/S0140-6736(20)30566-3 32171076 PMC7270627

[44] Weiss P Murdoch DR. Clinical course and mortality risk of severe Covid-19. Lancet (London, England). (2020) 395:1014–5. 10.1016/S0140-6736(20)30633-4 32197108 PMC7138151

[45] Grasselli G Greco M Zanella A Albano G Antonelli M Bellani G . Risk factors associated with mortality among patients with Covid-19 in intensive care units in Lombardy, Italy. JAMA Intern Med. (2020) 180:1345–55. 10.1001/jamainternmed.2020.3539 32667669 PMC7364371

[46] Li X Xu S Yu M Wang K Tao Y Zhou Y . Risk factors for severity and mortality in adult Covid-19 inpatients in Wuhan. J Allergy Clin Immunol. (2020) 146:110–8. 10.1016/j.jaci.2020.04.006 32294485 PMC7152876

[47] Xu PP Tian RH Luo S Zu ZY Fan B Wang XM . Risk factors for adverse clinical outcomes with Covid-19 in China: a multicenter, retrospective, observational study. Theranostics. (2020) 10:6372–83. 10.7150/thno.46833 32483458 PMC7255028

[48] Abu-Farha M Al-Mulla F Thanaraj TA Kavalakatt S Ali H Abdul Ghani M . Impact of diabetes in patients diagnosed with Covid-19. Front Immunol. (2020) 11:576818. 10.3389/fimmu.2020.576818 33335527 PMC7736089

[49] Cummings MJ Baldwin MR Abrams D Jacobson SD Meyer BJ Balough EM . Epidemiology, clinical course, and outcomes of critically Ill adults with Covid-19 in New York City: a prospective cohort study. Lancet (London, England). (2020) 395:1763–70. 10.1016/S0140-6736(20)31189-2 32442528 PMC7237188

[50] Zhou Y Ge YT Shi XL Wu KY Chen WW Ding YB . Machine learning predictive models for acute pancreatitis: a systematic review. Int J Med Inform. (2022) 157:104641. 10.1016/j.ijmedinf.2021.104641 34785488

[51] Izcovich A Ragusa MA Tortosa F Lavena Marzio MA Agnoletti C Bengolea A . Prognostic factors for severity and mortality in patients infected with Covid-19: a systematic review. PLoS ONE. (2020) 15:e0241955. 10.1371/journal.pone.0241955 33201896 PMC7671522

[52] Vaid A Somani S Russak AJ De Freitas JK Chaudhry FF Paranjpe I . Machine learning to predict mortality and critical events in a cohort of patients with Covid-19 in New York City: model development and validation. J Med Internet Res. (2020) 22:e24018. 10.2196/24018 33027032 PMC7652593

[53] Hu C Liu Z Jiang Y Shi O Zhang X Xu K . Early prediction of mortality risk among patients with severe Covid-19, using machine learning. Int J Epidemiol. (2021) 49:1918–29. 10.1093/ije/dyaa171 32997743 PMC7543461

[54] Bolourani S Brenner M Wang P McGinn T Hirsch JS Barnaby D . A machine learning prediction model of respiratory failure within 48 hours of patient admission for Covid-19: model development and validation. J Med Internet Res. (2021) 23:e24246. 10.2196/24246 33476281 PMC7879728

