# Corrigendum: Prevalence and outcomes of pancreatic enzymes elevation in patients with COVID-19: a meta-analysis and systematic review

**DOI:** 10.3389/fpubh.2023.1201405

**Published:** 2023-05-04

**Authors:** You Zhou, Yu-Tong Ge, Xiao-Xi Yang, Qian Cai, Yan-Bing Ding, Liang-Hao Hu, Guo-Tao Lu

**Affiliations:** ^1^Pancreatic Center, Department of Gastroenterology, Affiliated Hospital of Yangzhou University, Yangzhou University, Yangzhou, China; ^2^Yangzhou Key Laboratory of Pancreatic Disease, Institute of Digestive Diseases, Affiliated Hospital of Yangzhou University, Yangzhou University, Yangzhou, China; ^3^Department of Nursing, Affiliated Hospital of Yangzhou University, Yangzhou University, Yangzhou, China; ^4^Department of Oncology, Nanjing First Hospital, Nanjing Medical University, Nanjing, China; ^5^Department of Gastroenterology, Changhai Hospital, The Second Military Medical University, Shanghai, China

**Keywords:** COVID-19, pancreatic enzymes, elevation, outcome, meta-analysis, review

In the published article, there was an error regarding the affiliation(s) for [You Zhou]. As well as having affiliation(s) [1,2], they should also have [Department of Nursing, Affiliated Hospital of Yangzhou University, Yangzhou University, Yangzhou, China].

The affiliation for [Liang-Hao Hu] was still [Department of Gastroenterology, Changhai Hospital, The Second Military Medical University, Shanghai, China], the number of which should be changed to affiliation [5].

The authors apologize for this error and state that this does not change the scientific conclusions of the article in any way. The original article has been updated.

