# Supplementary material for: Prevalence and Outcomes of Pancreatic Enzymes Elevation in Patients With COVID-19: A Meta-Analysis and Systematic Review
Source: Front Public Health. 2022 May 12;10:865855. doi: 10.3389/fpubh.2022.865855 (PMC9133915; doi:10.3389/fpubh.2022.865855)
Supplement: Supplementary file 1 [file Table_1.DOCX]

Supplementary Material

# Supplementary Figures

**Supplement Figure 1.** Effect size analysis for the prevalence of AP in patients with PE elevation > 3× ULN

**
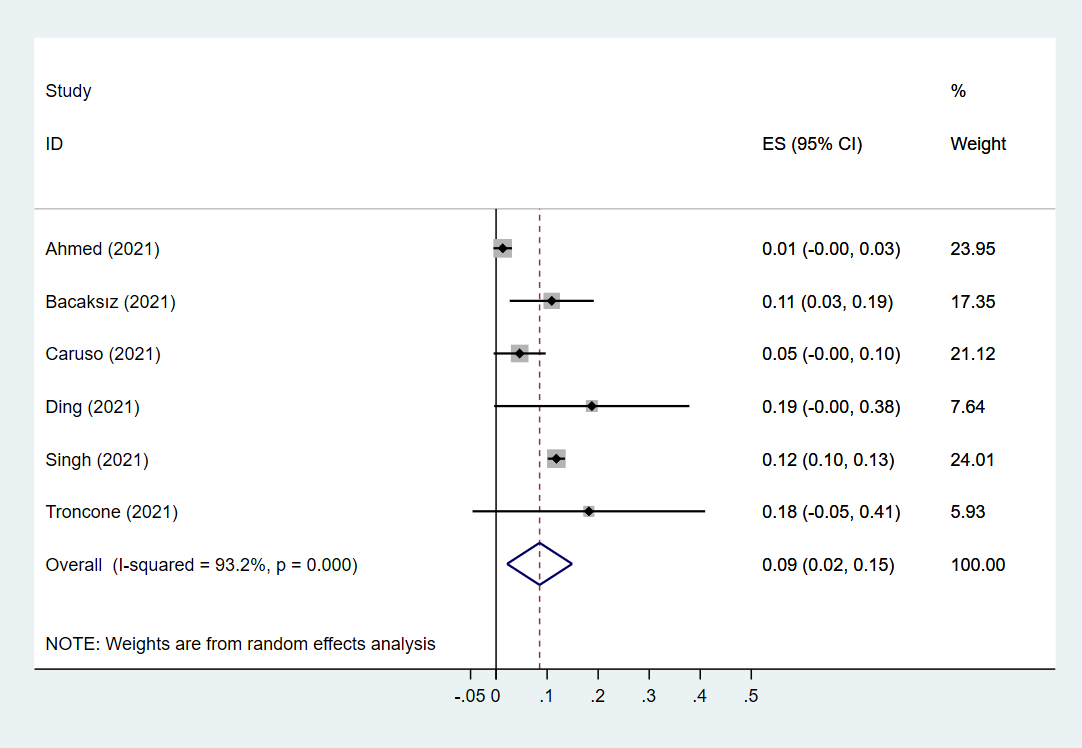
**

**Supplement Figure 2.** Sensitivity analysis of the mortality in COVID-19 patients with PE elevation

**
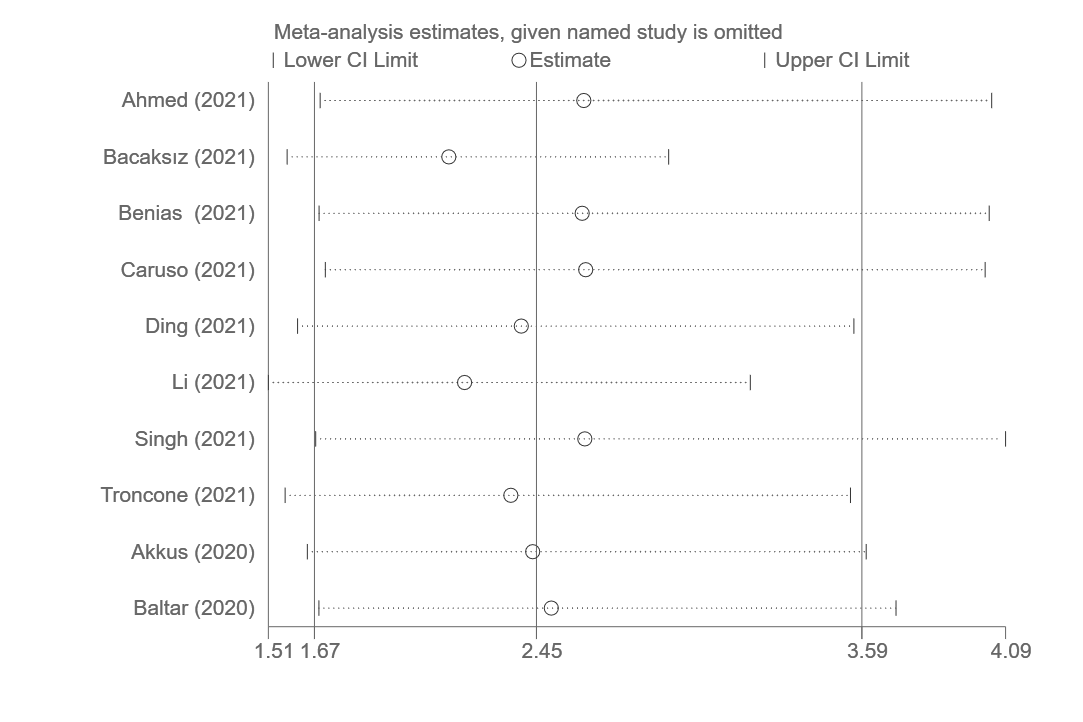
**

**Supplement Figure 3.** Effect size analysis for mortality in COVID-19 patients with PE elevation after removing heterogeneous studies

**
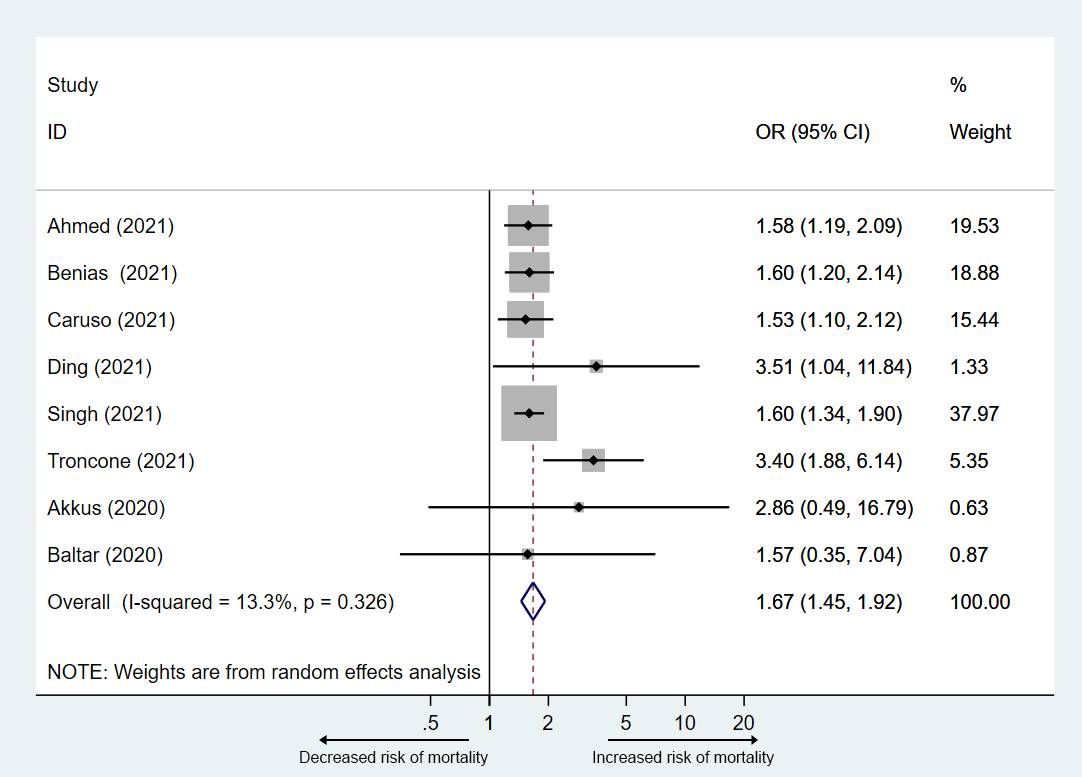
**

**Supplement Figure 4.** Effect size analyses for mortality in subgroup analysis of (A) 1-3 ULN group, (B) > 3× ULN group, (C) 1-3 ULN group after removing heterogeneous studies, (D) > 3× ULN group after removing heterogeneous studies, (E) single-center group, and (F) multi-center group

**
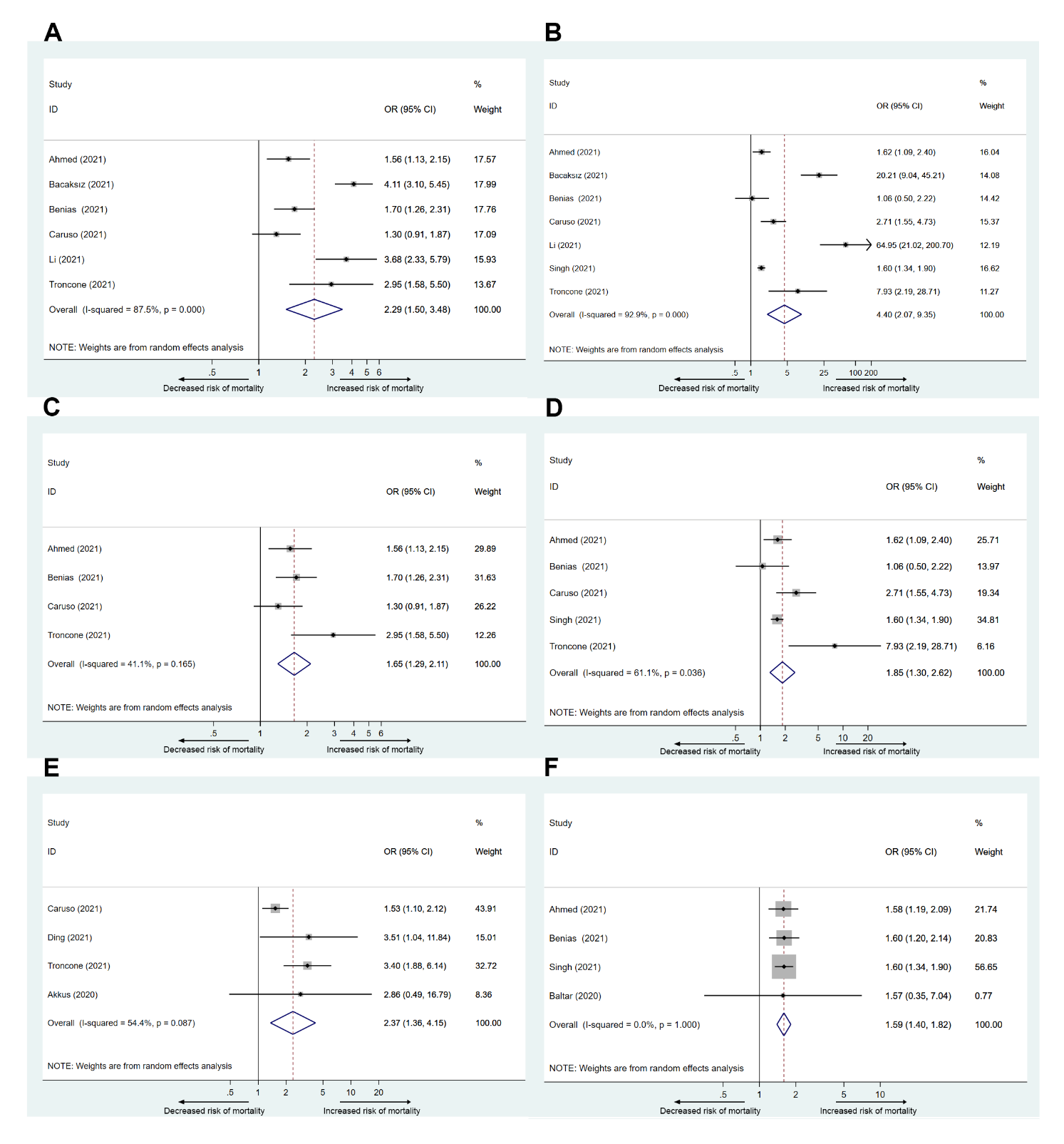
**

**Supplement Figure 5.** Effect size analyses for the association of PE elevation with (A) kidney injury, (B) respiratory failure, and (C) liver failure in COVID-19 patients


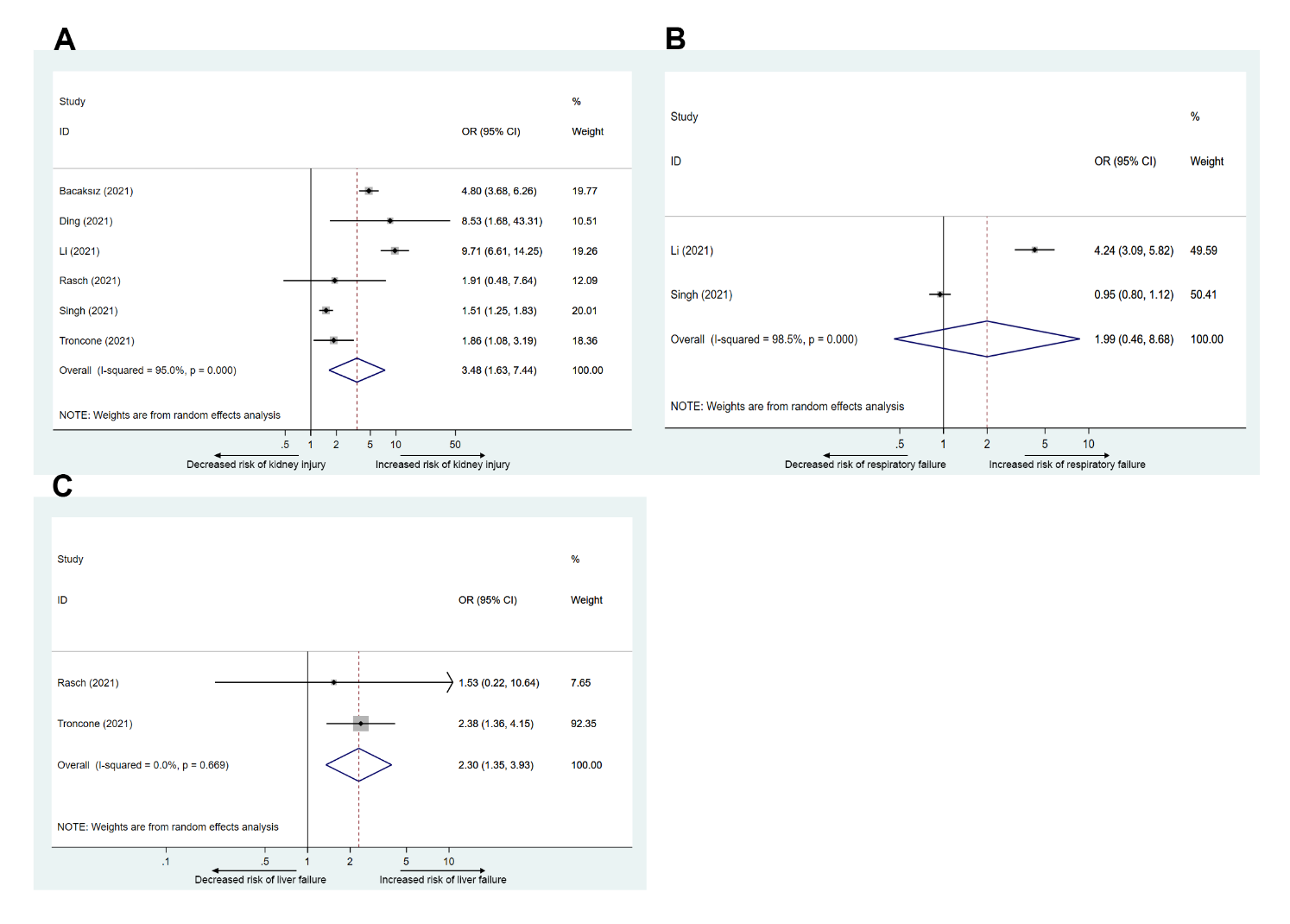


**Supplement Figure 6.** Funnel plots of studies reporting (A) the prevalence of PE elevation, and (B) mortality (C) ICU admission and (D) kidney injury in COVID-19 patients with PE elevation


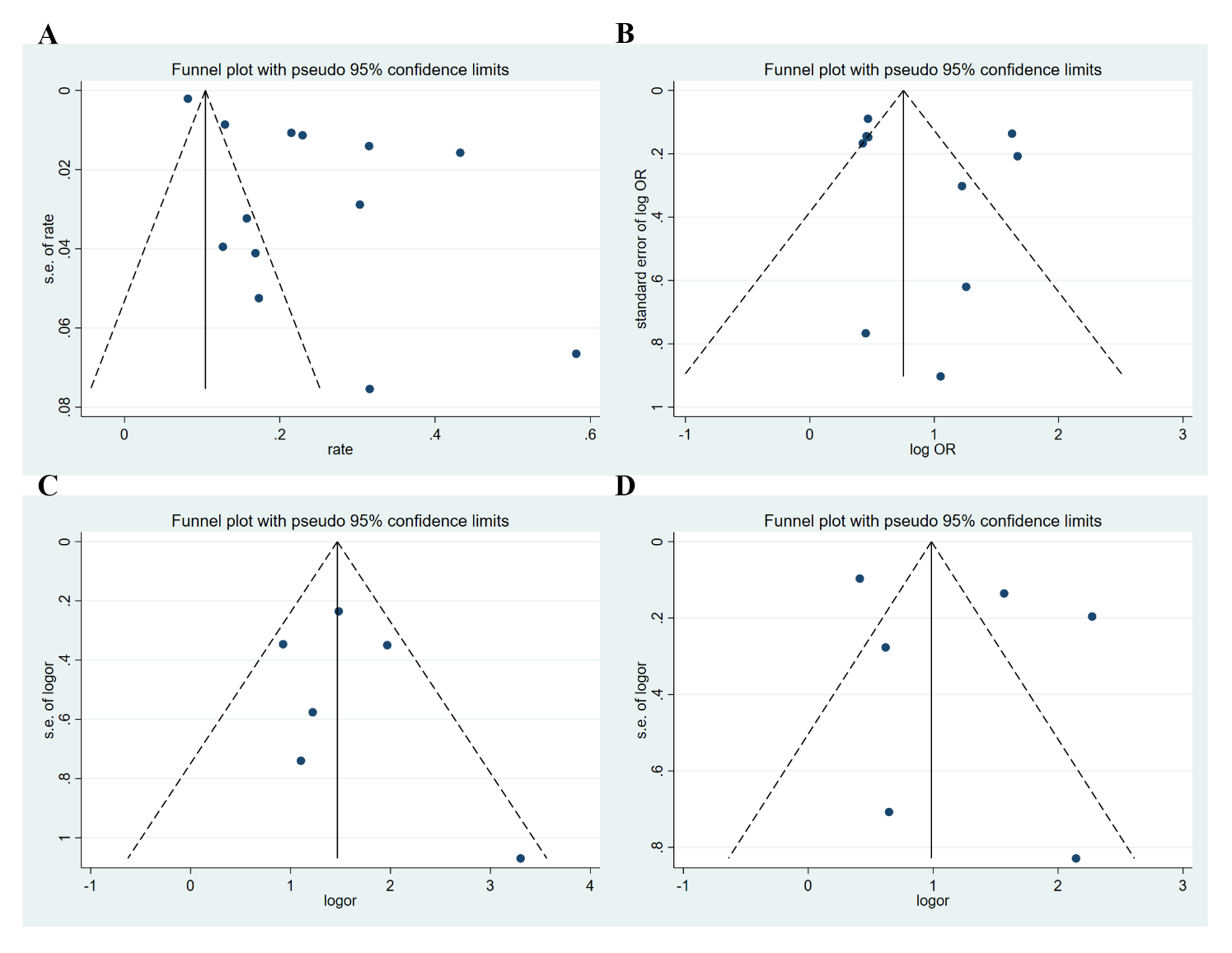


# Supplementary Tables

**Supplement Table 1.** Quality assessments of all included studies (n= 13) using the Quality in Prognostic Studies tool.

| Study | Study participation | Study attrition | Prognostic factor measurement | Outcome measurement | Study confounding | Statistical analysis and reporting |
| --- | --- | --- | --- | --- | --- | --- |
| Ahmed (14) | Low risk | High risk | Moderate risk | Low risk | High risk | Moderate risk |
| Bacaksız (13) | Moderate risk | Low risk | Low risk | Low risk | Low risk | Low risk |
| Benias (29) | Low risk | High risk | Moderate risk | Low risk | Low risk | Low risk |
| Caruso (21) | Low risk | Moderate risk | Moderate risk | Low risk | High risk | Moderate risk |
| Ding (22) | Low risk | High risk | Moderate risk | Low risk | Low risk | Moderate risk |
| Li (23) | Low risk | Low risk | Low risk | Low risk | Moderate risk | Low risk |
| Rasch (24) | Low risk | Low risk | Low risk | Low risk | High risk | Moderate risk |
| Singh (25) | Low risk | High risk | Moderate risk | Low risk | High risk | Moderate risk |
| Troncone (26) | Moderate risk | Low risk | Low risk | Low risk | Low risk | Low risk |
| Akkus (27) | Moderate risk | High risk | Low risk | Low risk | Moderate risk | Low risk |
| Baltar (16) | Moderate risk | Low risk | Low risk | Low risk | Moderate risk | Moderate risk |
| Barlass (28) | Moderate risk | High risk | Low risk | Low risk | Low risk | Low risk |
| Wang (12) | Moderate risk | Low risk | Low risk | Low risk | Moderate risk | Moderate risk |
